# dECM restores macrophage immune homeostasis and alleviates iron overload to promote DTPI healing

**DOI:** 10.1093/rb/rbad118

**Published:** 2024-01-17

**Authors:** Ju Zhang, Ruijuan Si, Yu Gao, Hui Shan, Qi Su, Zujian Feng, Pingsheng Huang, Deling Kong, Weiwei Wang

**Affiliations:** Tianjin Key Laboratory of Biomaterial Research, Institute of Biomedical Engineering, Chinese Academy of Medical Sciences and Peking Union Medical College, Tianjin 300192, China; School of Nursing, Qingdao University, Ningde Road, Shinan District, Qingdao, Shandong, 266071, China; Cancer Hospital of Tianjin Medical University, North Huanhu West Road, Tianjin, China; Tianjin Key Laboratory of Biomaterial Research, Institute of Biomedical Engineering, Chinese Academy of Medical Sciences and Peking Union Medical College, Tianjin 300192, China; The Affiliated Hospital of Qingdao University, 16 Jiangsu Road, Shinan District, Qingdao, China; Tianjin Key Laboratory of Biomaterial Research, Institute of Biomedical Engineering, Chinese Academy of Medical Sciences and Peking Union Medical College, Tianjin 300192, China; Tianjin Key Laboratory of Biomaterial Research, Institute of Biomedical Engineering, Chinese Academy of Medical Sciences and Peking Union Medical College, Tianjin 300192, China; Tianjin Key Laboratory of Biomaterial Research, Institute of Biomedical Engineering, Chinese Academy of Medical Sciences and Peking Union Medical College, Tianjin 300192, China; Nankai Univerisy, Tianjin, 300071, China; Tianjin Key Laboratory of Biomaterial Research, Institute of Biomedical Engineering, Chinese Academy of Medical Sciences and Peking Union Medical College, Tianjin 300192, China

**Keywords:** Deep tissue pressure injury, hydrogel, immunomodulation, tissue repair

## Abstract

Due to its highly insidious and rapid progression, deep tissue pressure injury (DTPI) is a clinical challenge. Our previous study found that DTPI may be a skeletal muscle injury dominated by macrophage immune dysfunction due to excessive iron accumulation. Decellularized extracellular matrix (dECM) hydrogel promotes skeletal muscle injury repair. However, its role in polarizing macrophages and regulating iron metabolism in DTPI remains unclear. Here, porcine dECM hydrogel was prepared, and its therapeutic function and mechanism in repairing DTPI were investigated. The stimulus of dECM hydrogel toward RAW264.7 cells resulted in a significantly higher percentage of CD206^+^ macrophages and notably decreased intracellular divalent iron levels. In mice DTPI model, dECM hydrogel treatment promoted M1 to M2 macrophage conversion, improved iron metabolism and reduced oxidative stress in the early stage of DTPI. In the remodeling phase, the dECM hydrogel remarkably enhanced revascularization and accelerated skeletal muscle repair. Furthermore, the immunomodulation of dECM hydrogels *in vivo* was mainly involved in the P13k/Akt signaling pathway, as revealed by GO and KEGG pathway analysis, which may ameliorate the iron deposition and promote the healing of DTPI. Our findings indicate that dECM hydrogel is promising in skeletal muscle repair, inflammation resolution and tissue injury healing by effectively restoring macrophage immune homeostasis and normalizing iron metabolism.

## Graphical abstract

**Figure:**
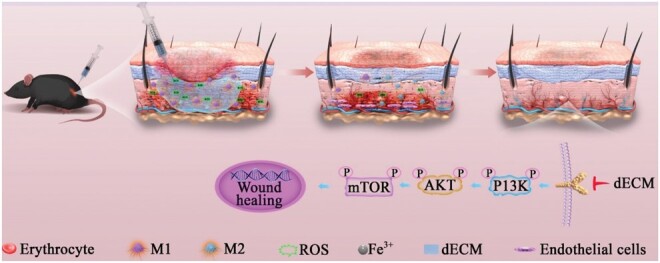

## Introduction

It is estimated that there will be between 20 and 60 million chronic wounds worldwide with an aging and obese population by 2026 [1]. Chronic wounds are the most pathogenic and fatal, including diabetic foot ulcers, chronic venous ulcers (CVU) and pressure injuries (PIs) [2]. PI, also known as bedsores or decubitus ulcers, and the annual cost of PI is estimated to be $1.7 billion in the USA alone [3]. Deep tissue pressure injury (DTPI) is a severe type of PI, which is prone to occur in the muscle tissue of the bone ridge. It is characterized by high insidiousness and rapid deterioration [4]. Therefore, researchers urgently need to reveal the pathological mechanisms and find precise therapeutic targets for early intervention and treatment of DTPI.

DTPI is a pressure-induced local microcirculatory disturbance and repetitive ischemia/reperfusion injury (I/RI) [5]. I/RI is a significant cause of cell death and organ damage in several pathologies, including myocardial infarction, stroke and acute renal failure [6–8]. Clinical studies have shown that iron overload occurs in I/RI [9]. Our previous study also found an increased level of free iron in the DTPI tissue [10]. Excess-free iron can interfere with wound healing by promoting the Haber–Weiss and the Fenton chemical reaction. It can induce a generalized inflammatory response and produce large amounts of reactive oxygen species (ROS) [11]. Based on the pathological characteristics of I/RI, we hypothesized that the rapid progression may be related to the iron overload of DTPI.

Macrophages are essential for maintaining iron homeostasis and repairing tissue [12]. In macrophages, genes associated with iron homeostasis are differentially expressed. M1-type macrophages mainly express ferritin, which is an iron storage protein. In contrast, M2-type macrophages mainly express iron transport proteins mediating iron release [13, 14]. An iron release can promote the growth of neighboring cells and the repair of tissues [13]. On the other hand, iron overload can also directly affect macrophages’ polarization and recruitment capacity [15]. Iron overload-induced sustained activation of pro-inflammatory M1-type macrophages in chronic wounds and increased local or systemic iron regulatory protein expression, severely disrupting tissue repair [16]. Therefore, immune regulation of macrophage polarization may improve iron metabolism and promote muscle repair of DTPI.

Extracellular matrix (ECM) is essential for the modulation of macrophage phenotype to enhance tissue repair [17]. Skeletal muscle-derived decellularized extracellular matrix (dECM) hydrogel is a promising material for muscle repair. dECM retains a native-like composition and can be injected and filled with irregularities [18]. In the peripheral arterial disease model, local injection of porous dECM hydrogel significantly promotes endothelial cell infiltration, arteriogenesis, muscle cell proliferation and muscle precursor cell infiltration [19, 20]. Researchers investigated the function of the immunoregulatory porcine brain macrophage-dECM in the spinal cord injury model. They found that the dECM hydrogel promoted the polarization of macrophages to the M2 phase and the outgrowth of neurites in cortical and hippocampal neurons. Porcine brain dECM promotes functional recovery in spinal cord injury models by activating M2 macrophages [21]. However, the role and critical mechanisms of dECM hydrogels in DTPI are still under investigation.

In summary, several studies have reported using dECM hydrogels for tissue repair. Theoretically, dECM hydrogels may be an effective treatment to promote DTPI. However, the therapeutic effect and mechanism of dECM in DTPI are not reported. In addition, the bioactive components of the protein in dECM have yet to be identified. This study used ICP-MS, flow cytometry and high-throughput genetic screening to identify iron overload and abnormal polarization of macrophages in the DTPI microenvironment. The dECM hydrogels, prepared by a chemical extraction method, have excellent biological properties and can promote cell adhesion and proliferation. The physicochemical properties of the biomaterial dECM were characterized. *In vitro* and *in vivo* experiments were performed to evaluate the immunomodulatory properties of macrophage polarization and iron metabolism and the vascular regenerative capacity of dECM hydrogels. The results suggest that dECM hydrogels may inhibit iron overload, which promotes DTPI-induced skeletal muscle regeneration, by regulating M2 macrophage expression through the P13K/Akt signaling pathway.

## Materials and methods

### Materials

Sodium dodecyl sulfate (SDS) was purchased from Aladdin (Shanghai, China). The deoxyribonuclease I, ribonuclease A and dsDNA test kits were purchased from Sigma-Aldrich (USA). SDS and Triton X-100 were supplied by Alfa Aesar (London, UK). The mouse ROS ELISA kit was purchased from FANKEW, Shanghai, China (no. F9261-A). Dulbecco’s modified medium (DMEM) and fetal bovine serum were purchased from Gibco Invitrogen (Waltham, MA, USA). Murine myogenic C2C12 cells were purchased from Science Cell (Carlsbad, CA, USA). RAW264.7 cells and culture medium were purchased from Wuhan Pnoxel Life Science & Technologies (no. CL-0122), and RAW264.7 cells were purchased from Wuhan Pnoxel Life Science & Technologies (no. CL-0190). Qingdao Yanshun Biotechnology Co. Ltd provided other organic reagents and experimental supplies.

### Fabrication of injectable hydrogel

Fresh skeletal muscle tissue from healthy adult pigs was collected at a slaughterhouse. The basic decellularization of porcine muscle tissue protocols were followed as previously published [22]. All steps were conducted with autoclave instruments and sterile solutions to fabricate sterile materials. Briefly, adipose tissue and fascia were isolated from porcine skeletal muscle and cut into small pieces (3–5 mm^3^). Sterilized distilled water was used to remove blood and impurities. It was shaken in 95% ethanol at 4°C for 12–24 h to remove lipids. This was followed by decellularization with 1% SDS and repeated rinsing and fluid change every 12 h until the tissue was completely white, usually 3–4 days. After the tissue turned white, the sterilized distilled water was replaced several times and thoroughly rinsed to remove the residual SDS until no bubbles existed after 30 s of vigorous shaking. A constant temperature shaker at 4°C was used throughout the decellularization process. The decellularized tissue was frozen, lyophilized and milled for long-term storage at −80°C.

Pepsin was dissolved in 10 ml 0.1M HCl at 1 mg/ml. After completely dissolved, 300 mg ECM powder was added and digested for 48 h on a magnetic stirrer to form a 3% w/v hydrogel. On this basis, it is diluted into hydrogels with 2% and 1% concentrations. Following digestion, add 1 M NaOH and ×10 phosphate-buffered saline (PBS) to neutralize the hydrogel and adjust the pH to 7.4. dECM hydrogel solution adjusted to the appropriate pH can be temporarily stored at 4°C.

### Characterization of the dECM hydrogels

Fresh porcine skeletal muscle and dECM were fixed in 4% paraformaldehyde for 24 h at room temperature. Hematoxylin and eosin (H&E) staining was performed after routine deparaffinizing and xylene cleavage. Five fields were randomly selected for analysis. The degree of decellularization was evaluated by extraction of total DNA according to the instructions of the dsDNA assay kit. DNA was quantified using a NanoDrop 2000 spectrophotometer. dECM hydrogels were added into EP tubes at different concentrations for 10 min at 37°C. Scanning electron microscopy (SEM) was used to observe the cross-sectional structure of lyophilized, dehydrated and vacuum-dried dECM (Hitachi, X-650, Japan). The rheological properties were measured using a rheometer (Anton Paar, Austria). A sinusoidal stress with a maximum amplitude of 50 Pa was applied. The angular frequency was kept constant at 3 rad/s. The temperature was increased gradually from 15 to 45°C for 30 min. Mass analysis was used to investigate the swelling behavior of dECM hydrogels. The dECM hydrogels were immersed in PBS at 37°C for a certain period and weighed after the PBS solution had been removed from the surface of the hydrogel. Hydrogel swelling rate (SR) was calculated using the equation SR = (Wd − Wt/Wt) × 100%. The swollen mass was denoted Wd and dried hydrogel as Wt, respectively. Experiments were repeated three times under identical conditions.

### *In vivo* degradation experiments of hydrogels

Female Balb/c mice (*n* = 3), aged 7–8 weeks and weighing 16–18 g, obtained from Huafu Kang Biotechnology Co. Ltd. The activated esterified CY5 was mixed in the dECM hydrogel overnight under light protection, stirred thoroughly to mix and injected into the dorsal subcutaneous area of the mice via a syringe based on the amidation reaction between the amino group and the carboxyl group in the activated ester. Mice were anesthetized by isoflurane inhalation at specific time points on Days 0, 3, 7, 14 and 21 and scanned using *in vivo* imaging. Images were analyzed using Maestro software with the following main parameters: exposure time 600 ms, excitation/emission spectra 640–700 nm. Fluorescence signals were labeled as a percentage of the maximum recording value, which was decreasing over time.

### Cell activities and proliferation assay

For this experiment, dECM hydrogels were added to 96-well plates at 100 μl/well and divided into four groups (six wells/group): control, 1% w/v gel, 2% w/v gel and 3% w/v dECM hydrogel. Subsequently, murine myogenic C2C12 cells were inoculated into the 96-well plates at 1 × 10^4^ cells/well and incubated for 24, 48 and 72 h, respectively. CCK-8 solution was added to the plates and incubated at 37°C for 1 h. The absorbance was read at 450 nm using a multifunctional enzyme marker (Thermo Fisher Scientific). According to the dead and live staining kit protocol, 1 μl of PI solution and 1 μl of calcein (4 mM) were added to 1 ml of DMEM medium. After swirling and mixing, cells were incubated for 30–45 min at room temperature. Results were obtained by fluorescence microscopy (Leica, USA).

### DTPI mouse model

All animal experiments were approved by the Laboratory Animal Ethics Committee, Institute of Radiation Medicine, Chinese Academy of Medical Sciences (IRM-DWLL-2023136).

SPF-grade C57BL/6 male mice were purchased from Huafu Kang Biotechnology Co. Ltd (weight 20 ± 2 g, 8–10 weeks). The DTPI model was established as described in the literature [10, 23]. Animals were maintained at room temperature (25°C) and 55% relative humidity. Standard laboratory pellet chow and water were provided. Animals were randomly assigned to the control group (no treatment), the DTPI group (saline injection) and the experimental group (dECM hydrogel injection), five mice in each group. The backs of the mice were shaved, and the area close to the skin of the sciatic nerve was selected. The same magnet (12 mm in diameter, 5 mm in thickness, 2.4 g, 1000 gauss surface flux) was applied and released for 12 h. The wound and surrounding tissue were then collected. The weight was ∼300 mg, and the volume was ∼0.5 cm^3^. The collected tissues were partially fixed in 4% paraformaldehyde.

### Assessing oxidative stress

#### Determination of changes in intracellular Fe^2+^ levels

The experiment was divided into control, ferric ammonium citrate (FAC), 2% dECM group and dECM+FAC group (three wells/group). dECM hydrogel was added to a six-well plate at 200 µl/well and incubated at 37°C for 30 min. The mito-ferrofluorescence probe was added to RAW264.7 cells in 1 × 10^5^ wells for 12 h. The cells were incubated at 37°C for 30 min, washed three times with serum-free medium, and placed under an inverted fluorescence microscope to determine the fluorescence intensity.

#### Malondialdehyde and superoxide dismutase assay

As described, dECM hydrogels were added to 24 well plates at 200 µl/well and incubated at 37°C for 30 min. RAW264.7 cells were collected and rinsed in PBS. WST-8/enzyme working solutions were added to the cells by the instructions of the superoxide dismutase (SOD) assay kit (Beyotime Biotechnology, No. S0103). Cells were incubated at 37°C for 30 min and centrifuged at 1000 g for 10 min. The 96-well plates were then plated with 200 μl of the supernatant. Absorbance was measured at 450 nm using a full-wavelength microplate reader (Thermo Fisher Scientific). Treated RAW264.7 cells were lysed for 30 min and analyzed according to the instructions of the malondialdehyde (MDA) assay kit (Beyotime Biotechnology, no. S0103). MDA assay solution was heated at 100°C for 15 min, cooled in a water bath to room temperature, centrifuged at 1000 g for 10 min, and 200 μl supernatant was added to 96-well plates. Complete wavelength absorption was measured at 532 nm. The assays were repeated three times under the same conditions.

#### ROS levels in vivo

Take different groups of fresh tissues, add a certain amount of PBS (PH7.4), homogenize the sample with a homogenizer, centrifuge the sample at 3000 rpm for 20 min, collect the supernatant, and then detect the amount of ROS in the tissues according to the protocol of the ELISA. The concentration of the samples is calculated according to the standard curve（*n* = 5）.

### Polarizing macrophages and vascularization

dECM hydrogels were incubated in 6 well plates at 37°C for 30 min at 200 μl/well. Subsequently, RAW264.7 cells were seeded in the six-well plates at a density of 5 × 10^4^ cells. M1 positive control (50 ng/ml IFN-r inducing M1 type) and M2 positive control (20 ng/ml IL-4 inducing M2 type) were also prepared. M0 type surface characterization molecule: F4/80 M1 type: CD86; M2 type: CD206. Cells were gently scraped, collected and incubated with 50 µl CD16/32 blocking solution (1:50) for 15 min at 4°C. Cells were incubated with 50 µl antibody for 1 h at 4°C. The supernatant was discarded, and the cells were re-suspended in PBS. Cells were detected by flow cytometry and analyzed using FlowJo software. Experiments were repeated three times under identical conditions.

As previously described, precooled 48 well plates were coated with 150 μl dECM, and Matrigel melted overnight at 4°C. Plates were then incubated at 37°C for 30 min. Digested HUVEC were seeded on gel at a density of 1 × 10^5^/well. Serum-free medium was added, and five duplicate wells were prepared and incubated at 37°C for 20 h. The results were analyzed by inverse microscopy and Image J software.

### Myotube formation evaluation

These experiments were divided into blank control, FAC (300 µM), dECM and dECM+FAC (three mixed wells per group). The 2% w/v dECM hydrogels were added to six-well plates at 200 μl/well and incubated at 37°C for 30 min. C2C12 cells were inoculated into the 1 × 10^5^ wells of the six-well plates and cultured for 48 h. Total RNA was harvested and analyzed by qRT–PCR for transcriptomic changes of critical genes involved in myotube formation to investigate the effect of dECM hydrogel on myotube differentiation and fusion.

### Biological mechanism of the dECM hydrogel

#### Establishment of the DTPI mouse model

Mouse were randomized into control (no treatment), DTPI (saline injection) and experimental (dECM hydrogel injection) groups. After DTPI, 100 µi dECM hydrogel or saline was injected subcutaneously. This procedure was repeated on alternate days up to the 14th day (six animals per group at each time point).

#### Wound healing rate

Photographs of local wound areas were taken on Days 1, 3, 7, 11 and 14. The wound area size and the mean of the unhealed wounds were automatically calculated using ImageJ software (V1.8.0.112, NIH). The baseline wound size was defined as the ratio of the area of the healing wound to the area of the traumatized wound on Day 3. The following formula was used to calculate the baseline area:

Initial trauma area (%) = (trauma area at each time point/trauma area at Day 3) × 100%.

#### Histological analysis

Mice were sacrificed in different groups on Days 1, 3, 7, 11 and 14, and skin and skeletal muscle were harvested from within 1 cm^2^ of the injury site. Tissues were fixed in 4% paraformaldehyde, paraffin-embedded and sectioned. The sections were cut at a thickness of 5 μm. As described above, H&E and Prussian blue staining were performed. Pathologic changes of inflammatory cell infiltration and iron deposition were observed by light microscopy (×40 and ×200). Five selected fields of each section were quantitatively analyzed using ImageJ software.

In addition, the paraffin-embedded sections were deparaffinized and dehydrated in a graded series of alcohols. Sections were embedded in 0.5% Triton X-100 for 10 min. Sections were incubated with 5% goat serum for 30 min, followed by overnight incubation at 4°C with various primary antibodies CD206 (Abcam, no. ab64693), CD86 (Abcam, no. ab239075), CD31 (Abcam, no. ab222783) and α-SMA (Abcam, no. ab240654) (1:100). Slides were washed three times with PBS for 5 min each and sealed with a fluorescence-quenching sealer. Images were observed with inverted fluorescence microscopy. Five images from each group were randomly selected for analysis. The fluorescence’s integrated gray value was calculated using the Image Pro Plus 9.0 image analysis software.

#### High-throughput microarray transcriptome analysis

Total RNA was extracted from tissues with Trizol reagent. After quality control, the mRNA was subjected to purification and fragmentation. Probe libraries were constructed by end repair, ligation and PCR amplification. Raw data were generated using an Illumina Novaseq™ 6000 high-throughput sequencer (LC-Bio Technology CO., Ltd, Hangzhou, China). Thus, Gene ontology (GO) functional enrichment analysis and Kyoto Encyclopedia of Genes and Genomes (KEGG) pathway enrichment analysis were performed by different groups. Lianchuan BioscienceTech (Hangzhou, China) performed the computer-assisted sequencing and bioinformatics analysis.

#### Reverse transcription quantitative PCR (RT-qPCR)

Total RNA samples were extracted according to the kit manufacture’s instructions (DP424, Tiangen, China). We calculated the relative mRNA expression of CD163, interleukin-10 (IL-10), tumor necrosis factor (TNF), inducible nitric oxide synthase (iNOS), arginase1 (Arg1), myosin heavy chain 8 (Myh8), myosin 10 (Myo10), myomesin3 (Myom3), myosin heavy chain 14 (Myh14), ferritin heavy chain1 (Fth1), solute carrier family 40 (slc40a1), ferrochelatase (Fech). The target gene’s relative expression was calculated using β-actin as the internal reference. The expression was repeated three times under the same conditions.

Kinsray Biotechnology (Nanjing, China) designed and synthesized the primers in *Table* 1.

#### Western blotting

Tissue samples were collected for protein concentration determination following the BCA kit instructions. After electrophoresis, PVDF was blotted with 5% BSA blocker to detect mouse polyclonal antibodies (1:100) against P13K (Cell Signaling, no. 4249), Akt (WL0003b), p-Akt (WLP001a), Hif-1a (WL01607), mTOR (WL02477) and p-mTOR (WL03694). The horseradish peroxidase-conjugated rabbit anti-mouse (1:5000) IgG-HRP secondary antibody was added to the slides. Films were scanned, and optical density values of target bands were analyzed using a gel imaging system (Gel-Pro-Analyzer software).

### Statistical analysis

Data are expressed as mean±SD. Statistical analysis was performed with the SPSS 25.0 software and the GraphPad Prism 7.0 software. Statistical differences were measured by Student’s *t*-test and one-way analysis of variance (ANOVA). Statistical significance was defined as a difference of *P* < 0.05.

## Results

### Properties and bioactive molecule content of dECM hydrogels

**Figure 1.**
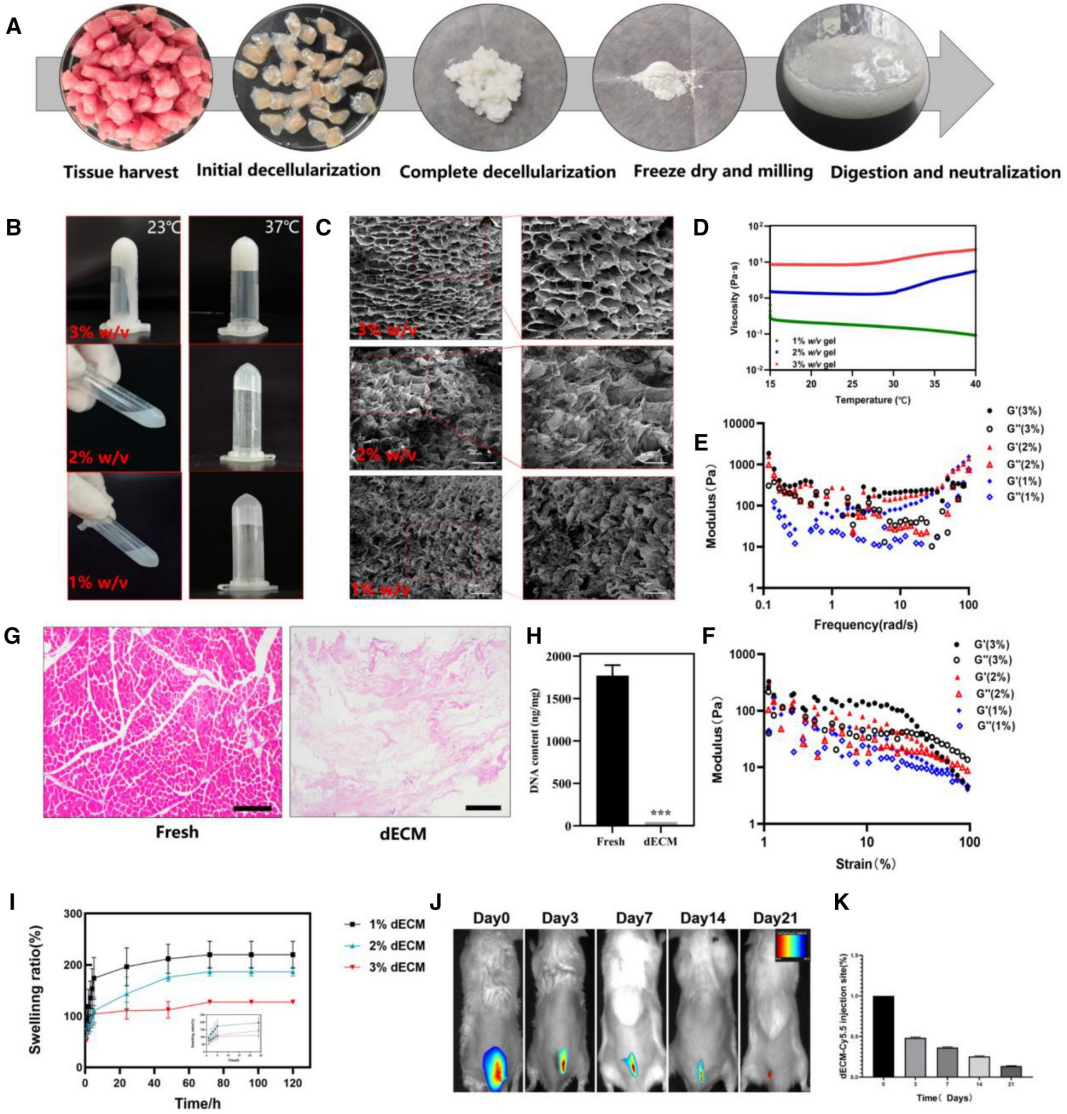
Process of characterizing and analyzing dECM hydrogel. (**A**) Preparation procedure of dECM hydrogels. (**B**) Gel formation photographs of dECM hydrogels with different concentrations between at 23°C and 37°C (1 wt/vol, 2 wt/vol and 3 wt/vol). (**C**) Structures of dECM hydrogel were observed by SEM. (**D–F**) Rheological analysis of the colloidal state, viscosity and shear rate of the dECM hydrogel. (**G, H**) H&E staining and DNA content of fresh muscle tissue and dECM. (**I**) Swelling property of dECM hydrogel in PBS. (**J**) Representative fluorescence images of dECM labeled by Cy5.5 at the injection site. (**K**) The percentage of fluorescence signals over time. Means± SD, ***P < 0.001 and ****P < 0.0001 by one-way ANOVA with Dunnett’s *t*-test, *n* = 3.

During dECM hydrogel formation, it can be observed that skeletal muscle tissue is transparent at the edges. The center of the tissue is slightly pink at the beginning of the decellularizing process and white after the ECM is completely decellularized (Figure 1A). We have investigated the gelation properties of different concentrations of the dECM powder. The dECM powder was digested with pepsin and hydrochloric acid to neutral pH of 7.4. The results showed that 1% w/v, 2% w/v and 3% w/v hydrogels were immobile and gelatinous after 10 min at 37°C. SEM images showed that different concentrations of dECM hydrogels all exhibited a similar nanofibrous network architecture (Figure 1B and C).

Meanwhile, viscosity gradually improved with increasing hydrogen concentration. The 1% w/v hydrogel had the lowest viscosity, and the hydrogel was more prone to leakage from the tissue after injection. The 3% w/v hydrogel had a higher resistance to injection (Figure 1D and F). We selected the 2% w/v hydrogel for the following experiments. The dECM muscle fiber structure was completely removed with no nuclei remaining, as shown by H&E staining. In contrast, the new skeletal muscle was intact and distributed in a regular fashion (Figure 1G). DNA content was reduced by over 90% in dECM compared to fresh tissue (Figure 1H, *P* < 0.001). Furthermore, Figure 1I shows the swelling characteristics of the dECM hydrogels in PBS. At 37°C, the SR of the dECM hydrogel gradually increased with time, and the equilibrium swelling was reached at 96 h. The equilibrium SRs of the dECM hydrogel were 219.78%, 186.68% and 127.30%, respectively. As the concentration increased, the equilibrium SR of dECM hydrogel decreased, which may indicate low degradation of internal ECM proteins. Protein molecular weight is high, which may indicate a low SR. The *in vivo* degradation of the gel was monitored using *in vivo* imaging in mice at various fixed time points, as shown in Figure 1J. It can be seen that the hydrogel degrades slowly *in vivo* as the imaging fluorescence signal of the subcutaneously injected hydrogel gradually decreases over time. This is due to the high sensitivity of the dECM proteins to enzymatic degradation.

**Figure 2.**
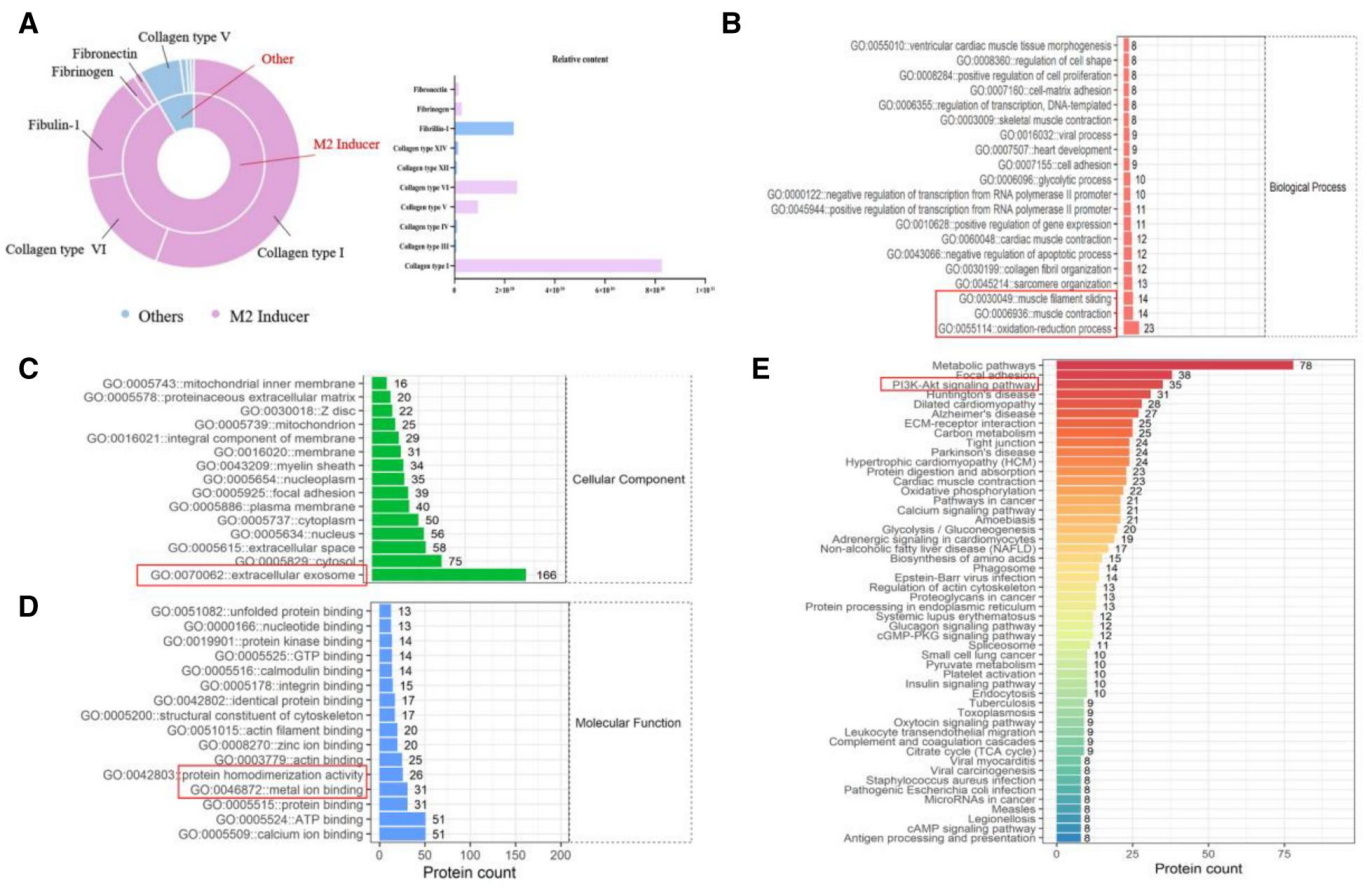
Proteomic analysis of the dECM hydrogel. (**A**) The proportion of the biological molecules in the dECM hydrogel. (**B–D**) The biological processes, components and molecular functions of dECM hydrogels, respectively (**E**), the major signaling pathways that are enriched in the dECM hydrogel.

The proteomic analysis revealed that 491 parental proteins were present in the dECM hydrogels. High levels of parental molecules were detected in dECM (Figure 2A), including type I collagen (ranked first in relative abundance) and fibronectin (known to induce M2 polarization). Functional enrichment analysis revealed that the components of the dECM hydrogel were mainly concentrated in the exosomes and the extracellular space, with a small intracellular (nuclear) fraction. Available molecular analyses revealed that these components were mainly involved in metal ion and ATP binding, protein binding, activity regulation and cytoskeletal composition and may be responsible for disrupting metal ion homeostasis in skeletal muscle. Further biological function analysis revealed that the potential functions of the dECM hydrogel were significantly involved in redox processes, skeletal muscle contraction and collagen fiber composition. KEGG pathway was enriched for the P13K-Akt pathway (Figure 2B and E) (*P* < 0.05).

### dECM hydrogels show good biocompatibility

**Figure 3.**
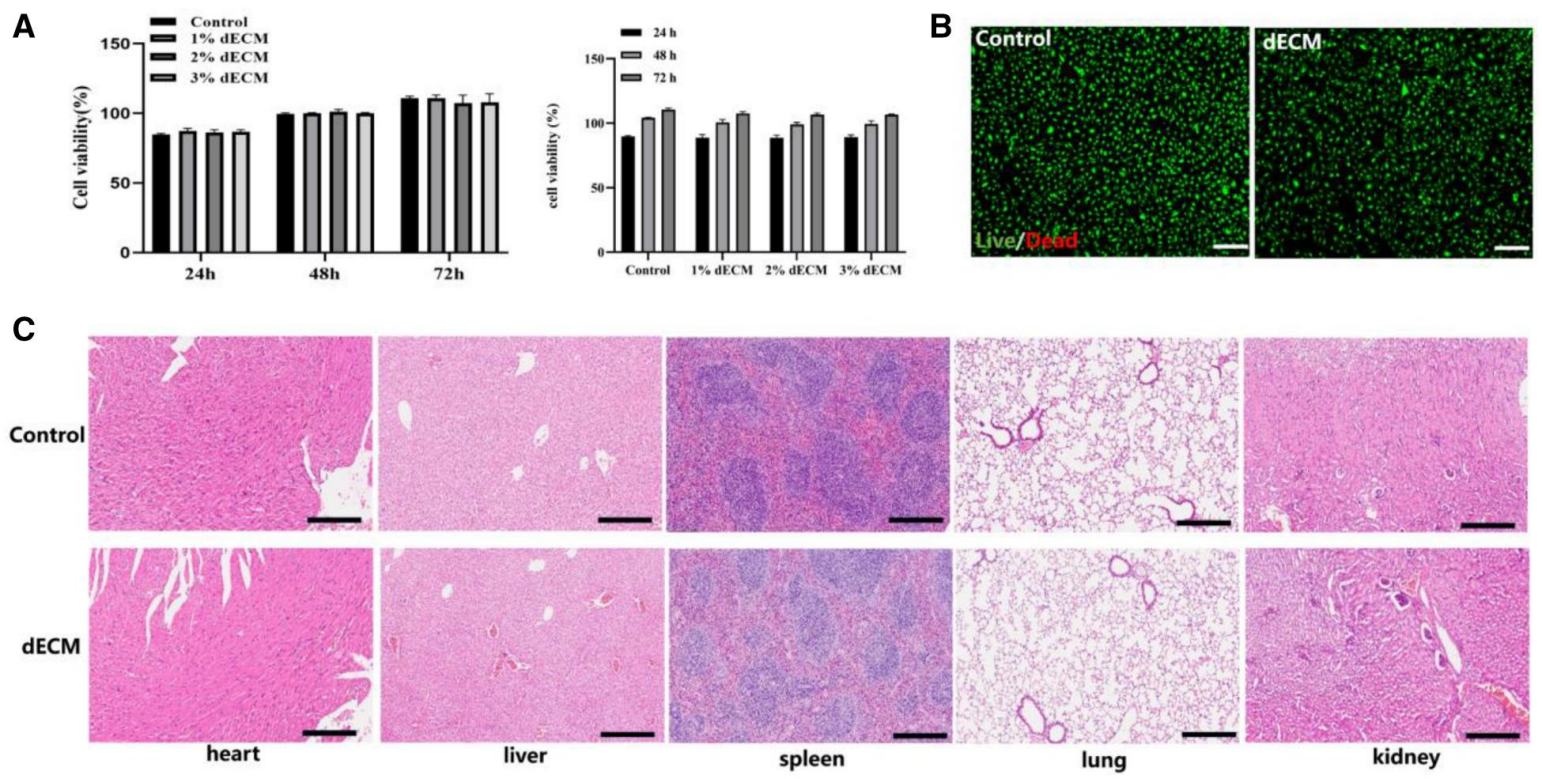
Biocompatibility of dECM hydrogels. (**A**) CCK-8 was used to analyze C2C12 cell proliferation in dECM hydrogels (1% w/v, 2w/v and 3w/v) at 24, 48 and 72 h. (**B**) Dead and live staining was used to analyze the survival of C2C12 cells in 2% dECM hydrogels. (**C**) H&E staining analyzed pathological structural changes of heart, liver, spleen, lung and kidney after injection of dECM hydrogel into mice on Day 14 (*n* = 3).

CCK-8 results showed no significant difference in absorbance at different time points for different dECM concentrations (Figure 3A). The effects of dead/living staining were shown in (Figure 3B), where almost all cells cultured on the 2% w/v dECM hydrogels were alive (green for living, red for dead). Mice were treated with dECM hydrogel or saline for 14 days by local intraperitoneal injection. HE staining indicated that no dECM hydrogels caused any local or systemic toxicity, as no significant pathological changes were observed in the mice’s heart, liver, spleen, lung or kidney (Figure 3C).

### dECM hydrogel promotes the polarizing of M2 macrophages

**Figure 4.**
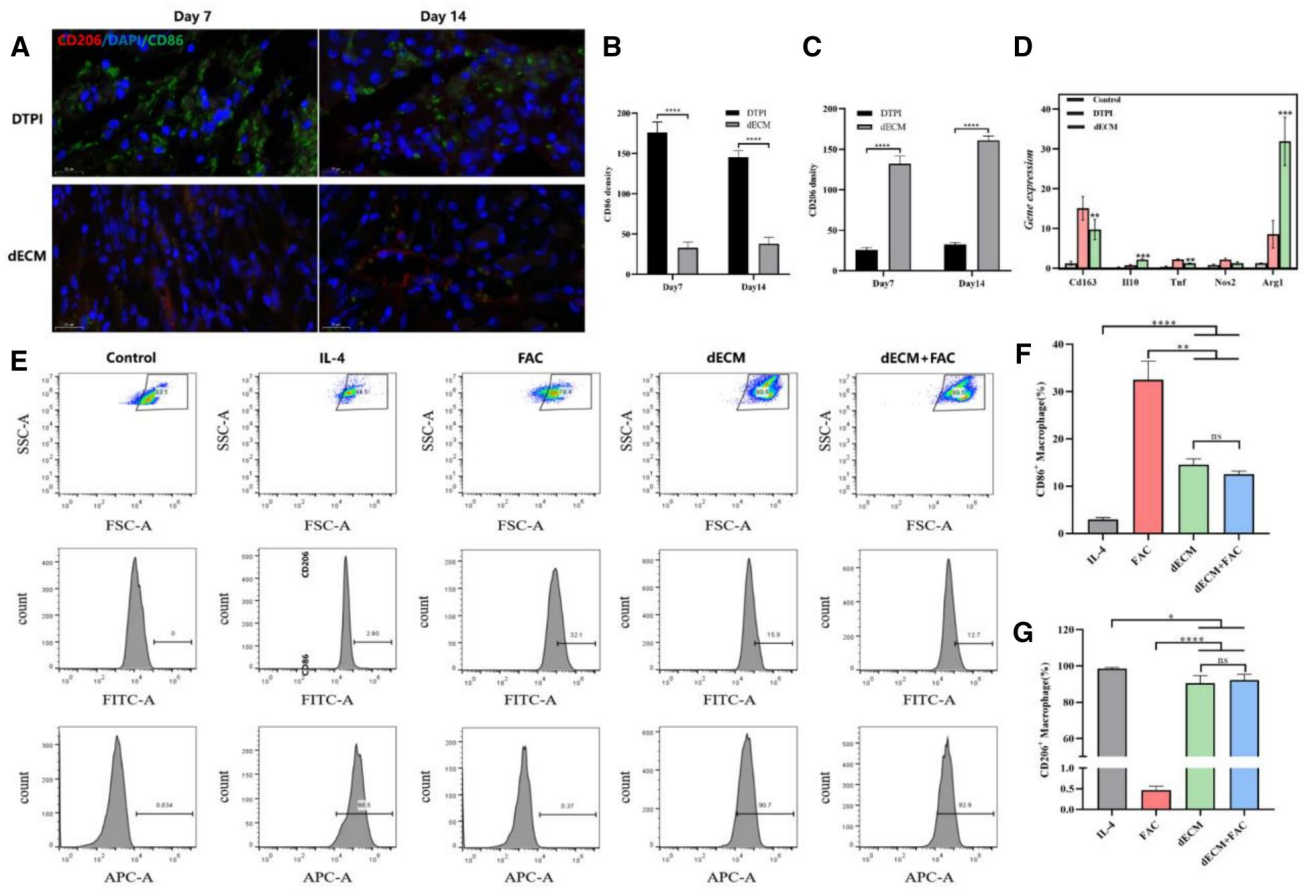
*In vitro* evaluation of the effect of dECM hydrogel on RAW264.7 macrophage polarization. (**A–C**) Immunohistochemistry for CD206 and CD86 expression in DTPI and dECM on Days 7 and 14. (**D**) Expression of macrophage-associated genes in different groups, including CD163, IL10, TNF-a, NOS2 and Arg1 (**E–G**). The effect of dECM on the polarity of RAW264.7 cells was analyzed by flowcytometry and the cells were divided into five groups (control, IL-4, FAC, dECM and dECM+FAC). M1 (CD86) and M2 (CD206). Mean±SD, *P < 0.05, **P < 0.01, ***P < 0.001, ****P < 0.001 and ****P < 0.0001 by 1-way ANOVA with Dunnett’s *t*-test, *n* = 3.

Immunofluorescence staining analysis revealed the induction of CD206^+^ cells in the dECM hydrogel compared to the control. The number of CD206^+^ cells was significantly higher in dECM than in DTPI on Days 7 and 14 (94.21 ± 6.02 vs. 30.67 ± 5.34, 118.01 ± 0.81 vs. 35.02 ± 0.62, *P* < 0.05) (Figure 4A). qRT–PCR analysis showed that iNOS and TNF-α (M1 markers) were significantly down-regulated in dECM compared to the control. However, Arg-1 (M2 marker) and IL-10 were significantly increased in dECM (*P* < 0.01). In addition, the expression of CD163 was significantly down-regulated (*P* < 0.05) (Figure 4B). Furthermore, the effect of dECM on macrophage polarization was determined in RAW264.7 cells. Flow cytometry results showed that after FAC (300 µmol/l) for 48 h, the percentage of CD86-positive cells was (32.1% ± 3.78%). The percentages of CD206 positive cells were (90.7% ±  1.40%) and (91.2% ±  2.27%) in the dECM and dECM + FAC groups, respectively. The two groups had no statistically significant difference (Figure 4C and D).

### dECM hydrogels inhibited oxidative stress and promoted vascularization and modularization

**Figure 5.**
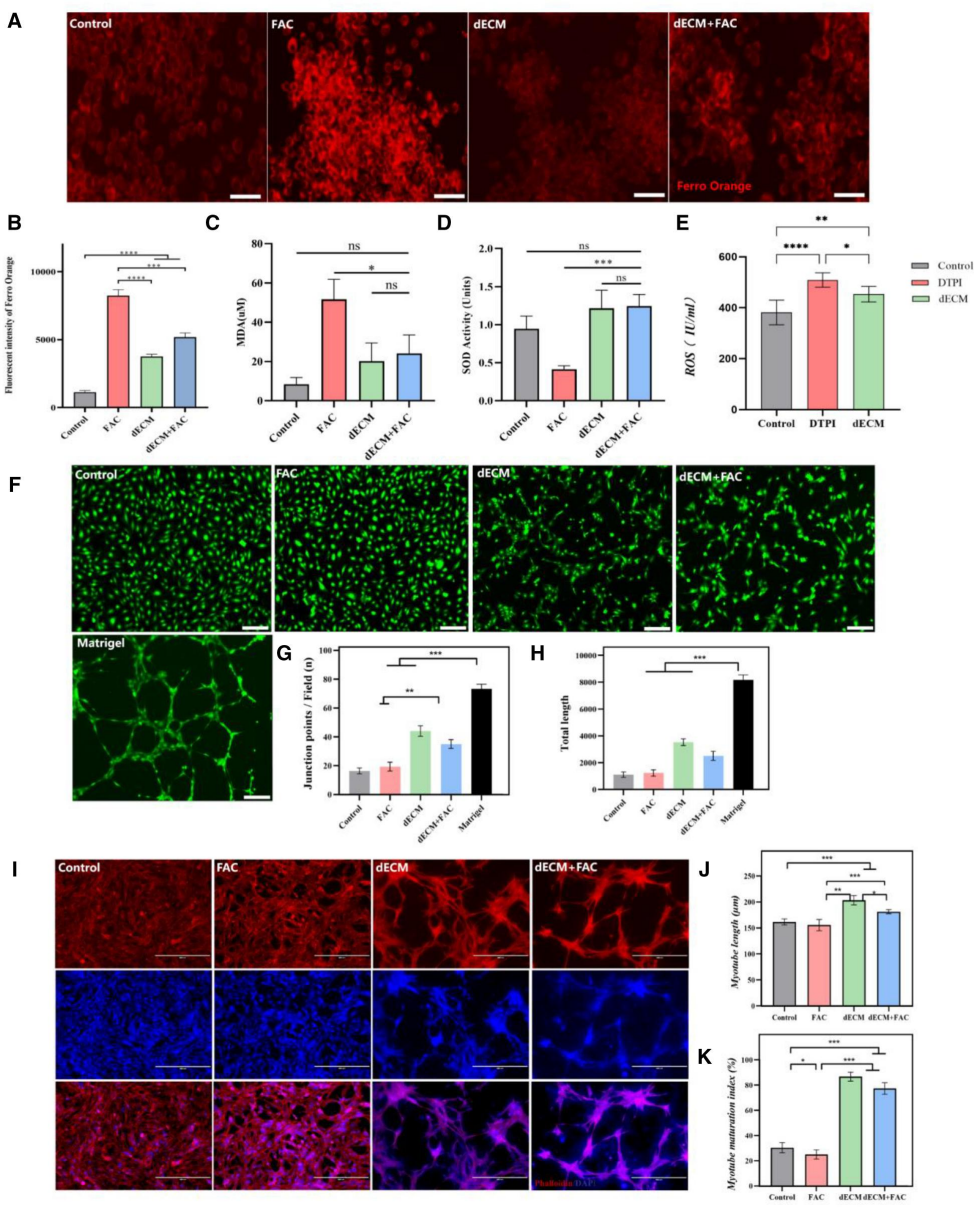
Evaluation of dECM hydrogels in oxidative stress, vascularization and myotubular differentiation. (**A, B**) Mito-Ferro Orange fluorescence density in RAW264.7 cells in the control group, the FAC group, the dECM group and the dECM+FAC group. (**C, D**). SOD and MDA levels were analyzed in RAW264.7 cells after 24 h. (**E**) ELISA was used to analyze the release of ROS in DTPI and dECM groups on Day 3. (**F–H**) The number and length of tubular nodules were analyzed in HUVEC cells, and Matrigel was used as a positive control. (**I–K**) Phalloidin staining was used to observe the morphology of the cytoskeleton of C2C12 cells in dECM hydrogels. Mean±SD, *P < 0.05, **P < 0.01, ***P < 0.001 and ****P < 0.0001 by one-way ANOVA with Dunnett’s *t*-test, *n* = 3.

The Mito-Ferro Orange fluorescence probe was used to detect Fe^2+^ changes in RAW264.7 cells. It was found that the intracellular Fe^2+^ concentration was increased in FAC groups compared to control groups (*P* < 0.001). Meantime, Fe^2+^ concentration was significantly decreased in dECM group (*P* < 0.001) (Figure 5A and B). MDA activity was increased in the FAC group after treatment of RAW264.7 cells with 300 μmol/l FAC for 24 h compared with the control group. In contrast, there was a significant decrease in MDA activity in the dECM hydrogel group (*P* < 0.001). In addition, there was an increase in SOD activity in the dECM group compared to the FAC group (*P* < 0.05) (Figure 5C and D). The concentration of ROS in the DTPI group was significantly higher than that in the control group and in the dECM group, with significant differences between the groups (*P* < 0.05) (Figure 5E). The dECM group significantly promoted the formation of lumen structure in HUVECs compared to the control group. Although less complete lumen formation was observed (*P* < 0.05) (Figure 5F and H). Phalloidin staining was performed to observe C2C12 morphological changes in dECM hydrogels. C2C12 cells of the control group had no apparent tubular structures. However, C2C12 cells in dECM and dECM+FAC groups exhibited different tube structures. The number of myotubes was significantly higher than the control (57.17 ± 4.12 vs. 21.04 ± 1.50) and (52.68 ± 2.14 vs. 21.04 ± 1.50) (*P* < 0.05) (Figure 5I–K).

### dECM hydrogel enhances DTPI wound healing

**Figure 6.**
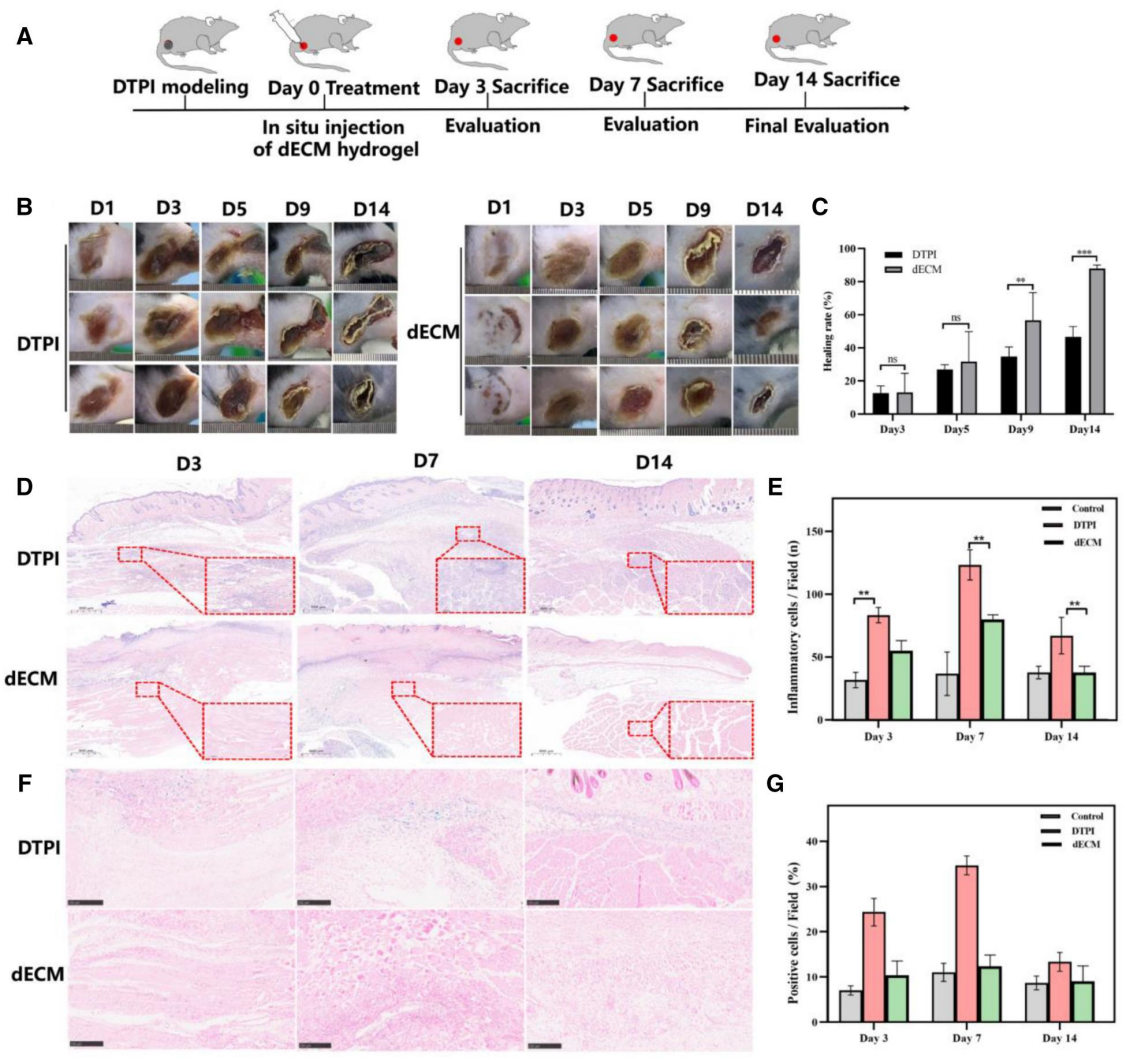
Evaluation of dECM hydrogels on DTPI healing. (**A**) Schematic of the treatment at different time points in DTPI mice. (**B, C**) Photographs of wound healing and wound rate statistics of DTPI and dECM on Days 1, 3, 7, 11 and 14, respectively. (**D, E**) H&E staining analysis of inflammatory cell infiltration in DTPI on Days 3, 7 and 14. (**F, G**) Prussian blue staining analysis of ion content of DTPI and dECM on Days 3, 7 and 14, arrows indicate iron.

**Figure 7.**
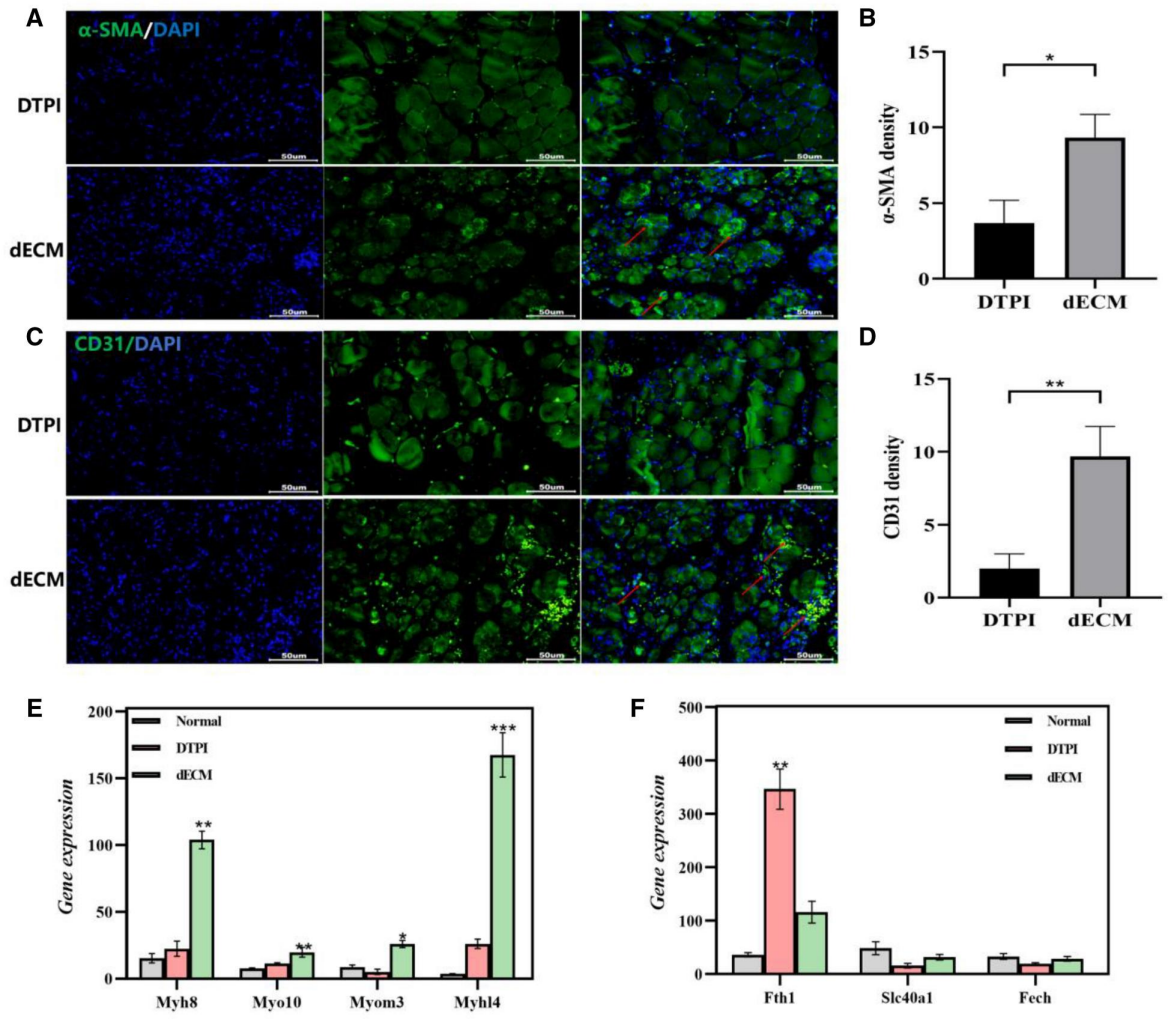
Evaluation the effects of dECM hydrogels on angiogenesis and iron metabolism (**A–D**) Immunofluorescence staining was used to analyze α-SMA and CD31 positive cell expression in DTPI and dECM mice on Day 14. (**E, F**) qRT–PCR was used to analyze iron metabolism-related genes including Slc40a1, Fech and Fth1 and skeletal myogenic genes including Myh8, Myo10, Myom3 and Myhl4 in DTPI and dECM groups on Day 14. Mean±SD, *P < 0.05, **P < 0.01, ***P < 0.001 and ****P < 0.0001 by one-way ANOVA and Dunnett’s *t*-test, *n* = 3.

A schematic view of the treatment at different time points is shown in Figure 6A. The wound healing rates in the dECM group were significantly higher than those in the control group on the ninth day. The wound size gradually decreased significantly on Day 14. This indicated that the dECM hydrogel effectively promoted DTPI wound healing (Figure 6B and C). Furthermore, H&E staining revealed significant inflammatory cell infiltration at the wound site and absence of prominent granulation tissue in the DTPI group (Figure 6D and E). Prussian blue staining revealed that the iron content of the tissues was significantly decreased after dECM treatment (*P* < 0.05) (Figure 6F and G). Paraffin sections of *in vivo* tissues were used to count the number and diameter of blood vessels in the injured area. Immunostaining results showed that CD31 and α-SMA were significantly increased in blood vessels and skeletal muscle in the dECM group on Day 14 compared with the control group (Figure 7A–D). qRT–PCR results showed that the vital iron metabolism genes Slc40a1 and recombinant ferrochelatase (Fech) were significantly increased in the dECM hydrogel compared with the control group (*P* < 0.05). At the same time, ferritin heavy chain 1 (Fth1) was significantly down-regulated in the dECM hydrogel compared to the control group (*P* < 0.01). Meanwhile, skeletal muscle genes Myh8, Myo10, Myom3 and Myl4 were significantly up-regulated in dECM hydrogel (Figure 7E and F).

### dECM hydrogel may activate the P13k/Akt pathway to improve DTPI repair

**Figure 8.**
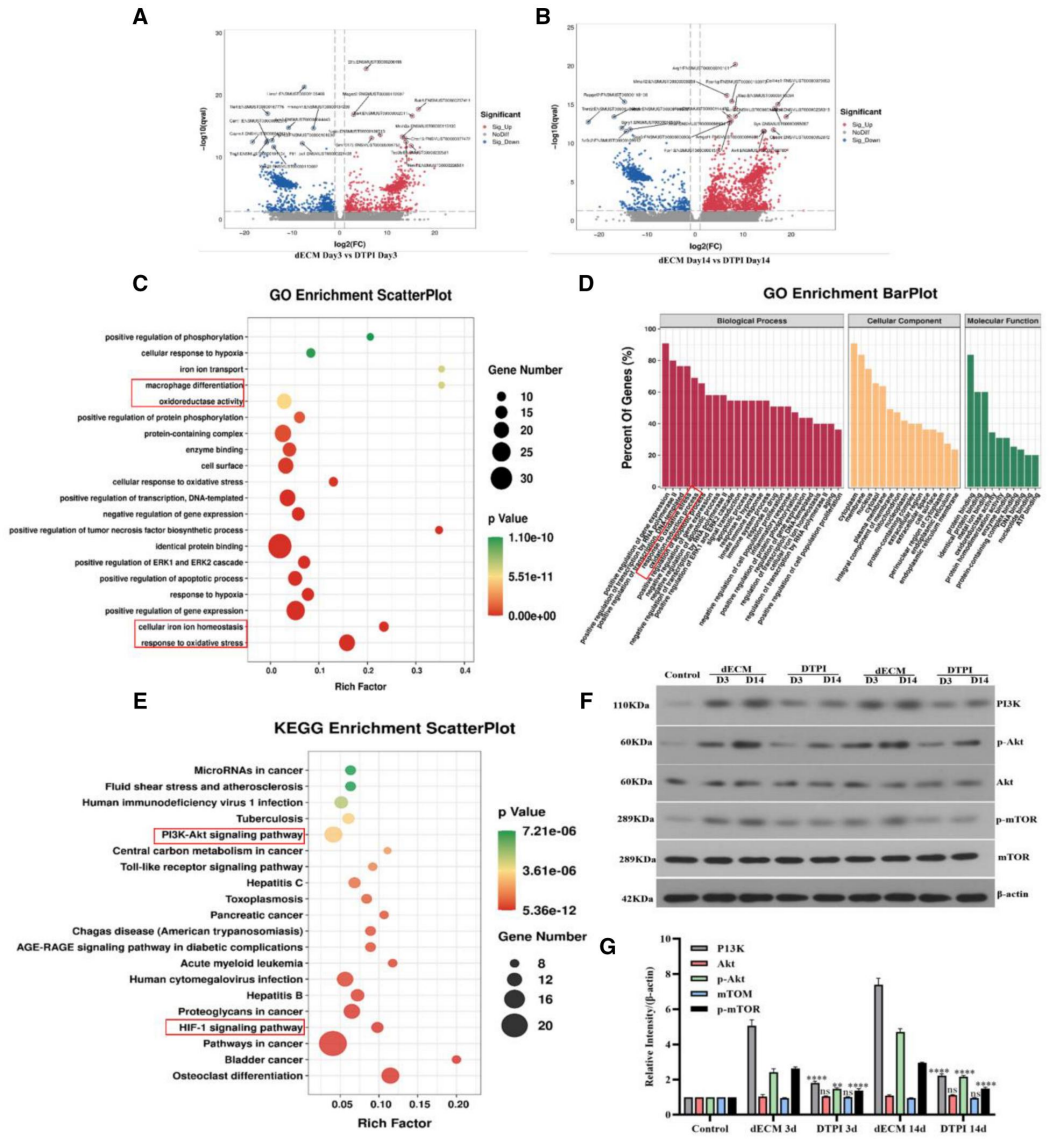
The mechanism of dECM to promote DTPI healing. (**A, B**) Volcano plots show the differential gene expression between the DTPI and dECM groups on Days 3 and 14. (**C–E**) GO and KEGG enrichment analysis of the biological function and the key signaling pathways of dECM *in vivo*. (**F, G**) Western blot analysis of p-Akt, Akt, P13K, p-mTOR and mTOR protein levels on Days 3 and 14 for Control, DTPI and dECM groups. Mean±SD, *P < 0.05, **P < 0.01, ***P < 0.001 and ****P < 0.0001 by one-way ANOVA with Dunnett’s *t*-test, *n* = 3.

The mechanism of dECM was investigated in detail by analyzing the transcriptome in DTPI. The results of the volcanic plots showed that 493 and 1127 genes were differentially expressed in dECM compared with the control on Days 3 and 14, respectively (false discovery rate <0.05) (Figure 8A and B). The results showed 26 112 differential genes were enriched in 1354 GO entries and 154 KEGG pathways in the dECM group compared to the control group on Day 3. On Day 14, there were 32 102 differential genes in the dECM group. These genes were enriched in 5382 GO entries and 273 KEGG pathways. GO analysis revealed 390 down-regulated and 103 up-regulated genes. These genes were associated with immune response, metabolism and redox processes on Day 3. Meanwhile, 978 genes were up-regulated, and 149 were down-regulated in the dECM group on Day 14. These genes included those involved in angiogenesis, skeletal muscle repair, proliferation and wound healing. KEGG enrichment analysis showed that dECM significantly affected the P13K/Akt-mTOR pathway. This pathway was related to apoptosis, cell proliferation, positive immune system regulation, cell proliferation, transcription and vascular regeneration (Figure 8C and E). Western blot results showed that protein expression levels of P13K, Akt, p-Akt, mTOR and p-mTOR were significantly down-regulated in dECM at Days 3 and 14 compared with control (*P* < 0.05) (Figure 8F and G).

## Discussion

In this study, the dECM hydrogel promoted the conversion of M1–M2 macrophages. It also reduced iron deposition. In addition, the dECM hydrogel enhanced vascular regeneration and skeletal muscle repair by inhibiting inflammatory cytokines during tissue repair. The results of the high-throughput transcriptome array indicated that dECM might be involved in the immune regulation of iron metabolism and the polarization status of macrophages through the P13k/Akt-mTOR signaling pathway to promote DTPI repair.

Previous studies have implicated iron accumulation in chronic wounds [24]. In 2008, researchers proposed: ‘Iron deposition is closely related to pathological changes in chronic venous ulcers (CVU)’. In CVU, iron deposition activates M1-type macrophages to produce excessive ROS, destroying and degrading dermal tissue [25]. Free iron in the wound exudate has also been reported in patients with diabetic foot ulcers. There was a negative correlation between serum iron and wound exudate iron levels in patients with diabetic foot ulcers [26, 27]. The iron deposition also induces sustained activation of pro-inflammatory M1 macrophages in patients with diabetic foot ulcers and promotes an increased expression of local or systemic iron regulators [16]. This study used ICP-MS and Prussian blue staining to analyze iron levels in DTPI tissues at different time points. The results showed an increase in iron levels with the progression of DTPI. In addition, we confirmed that M1 macrophages were hyperpolarized in the microenvironment of DTPI by flow cytometry. In conclusion, iron overload and over-activation of M1 macrophages are present in DTPI and other chronic wounds.

ECM hydrogels are essential in regulating the immune system [28]. Bovine bone ECM hydrogel is a biocompatible biomaterial that promotes fibroblast proliferation and induces macrophage pro-inflammatory cytokine production during tissue repair [29]. Therefore, the injectable bovine bone ECM hydrogel can dynamically integrate multiple biological functions at different stages of the fracture healing process [30]. In this study, the expression levels of iNOS (a marker specific for M1-type macrophages) and pro-inflammatory factors, such as TNF-a and IL-6 were abnormally increased in DTPI. The expression levels of CD206, Arg1 and Slc40a1 (ferroportin 1) were significantly up-regulated in the dECM group. These results indicated that the dECM hydrogel promoted the conversion of M1-type macrophages to M2-type macrophages.

On the other hand, the expression levels of M1-type macrophages (CD86 and NOS2) and the iron metabolism-related genes Fth and transferrin receptor 1 were found to be significantly down-regulated in the dECM group. These results suggest that dECMs improve iron metabolism by modulating macrophage heterogeneity. In conclusion, our results suggest that the amelioration of iron deposition in DTPI may benefit from ECM hydrogels’ heterogeneous immunomodulation of macrophages. In particular, the heterogeneity of macrophages is critically essential for muscle regeneration [31]. In the early stages of DTPI, the dECM hydrogel induces an inflammatory response from M1-type macrophages, which are pro-inflammatory, to M2-type macrophages, which have anti-inflammatory effects. During tissue remodeling, dECM not only promoted M2-type macrophage polarization to suppress inflammation but also promoted vascular endothelial cells and muscle regeneration. dECM effects on HUVEC and mouse C2C12 myogenic cells were evaluated *in vitro*. It was confirmed that dECM hydrogel enhanced the formation of tubular structures in HUVEC cells by calcein staining. Furthermore, in dECM hydrogels suitable for the culture of skeletal muscle-like cells, C2C12 cells formed sticky patches and filamentous pseudopods.

In addition, it has been shown that dECM hydrogels are beneficial in promoting angiogenesis and regenerating skeletal muscle [18]. In a previous study, dECM injection in a rat hindlimb ischemia model increased arteriolar and capillary density and recruitment of connexin- and MyoD-positive cells [19]. In DTPI, the dECM group was more effective in promoting angiogenesis, CD31 expression and skeletal muscle repair Myh8, MyO10, Myl4 and α-SMA expression in injured tissue 2 weeks after injury. In order to elucidate the biological mechanisms of dECM hydrogels, we performed a comprehensive analysis of their protein composition fractions by liquid chromatography-mass spectrometry. We found that porcine dECM hydrogels contained 491 parent macromolecules. The most important active components were collagen, fibronectin and adhesion proteins. The major functional components of dECM hydrogels have been ascribed to complicated biological functions. The alpha2beta1 peptide CB3 (Asp-Gly-Glu-Ala) fragment in type I collagen stimulates the upregulation of M2 macrophages [32]. Laminin alpha2 is mainly expressed in skeletal muscle and pericytes. Pericytes are involved in the attachment of the ECM to the cell surface. The binding to its receptor impacts the repair signaling in the skeletal muscle [33]. Bioinformatic analysis of the differential proteins indicated that they are involved in vascular regeneration, skeletal muscle repair, the immune response, oxidative stress and the binding of metal ions. In addition, the differential proteins were significantly enriched in the P13K-Akt pathway as determined by GO and KEGG enrichment analysis. The dECM hydrogel may ameliorate the early oxidative stress milieu by modulating the polarity of the macrophages.

We further analyzed the transcriptome profile of the tissues after the implantation of dECM hydrogels. To our knowledge, no reported *in vivo* transcriptome analyses of dECM hydrogels exist. In this study, we analyzed and validated the changes in genes involved in immune regulation expressed in dECM hydrogels. We found significant differences in gene expression among the three groups. The most important pathways included immune control, macrophage differentiation, oxidative stress, skeletal muscle repair, vascular regeneration and iron metabolism. The dECM hydrogel significantly increased the level of CD68 transcript in the macrophages of the DTPI group on Day 3. This suggests that early placement of the biomaterial may induce an acute inflammatory response and recruitment of high numbers of M1 macrophages and lay the foundation for macrophage polarity switching in subsequent tissue repair [34]. The dECM hydrogels significantly increased neovascularization and vascular endothelial growth factor at the injury sites, possibly through the secretion of pro-angiogenic factors by M2-type immune cells and possibly by releasing bioactive peptides.

Microarray analysis showed that the M2-type cell marker Arg-1 was significantly up-regulated in the dECM group. This was associated with increased pro-angiogenic factors and decreased expression of pro-inflammatory cytokines. This was consistent with optimizing the anti-inflammatory/immune microenvironment and stimulating the maximal angiogenic capacity. Whether the process of vascular regeneration was related to degrading active peptides was not studied. Iron metabolism was also affected by the polarization switch of the macrophages in a hypoxic environment [35]. Prediction pathway analysis revealed that iron metabolism was altered in DTPI. Specifically, Slc40a1 and Fech, two enzymes involved in transporting and neutralizing excess iron, were up-regulated on Days 3 and 14 after dECM injection. Furthermore, the inflammatory factors IL-1β, IL-6 and TNF-alpha transcript levels were significantly suppressed. Furthermore, expression levels of MyO and Myh genes, which are associated with skeletal muscle regeneration, were up-regulated in dECM. Thus, the implantation of dECM hydrogel played an essential role in angiogenesis and skeletal muscle regeneration.

Moreover, KEGG analysis showed that the PI3K/Akt pathway was highly enriched in the dECM hydrogel group. PI3K/Akt is an essential human signaling pathway that controls key tissue repair processes, including cell proliferation and angiogenesis [36]. Previous studies have shown that growth factors, including VEGF and TGF-1β, activate PI3K/Akt [37, 38]. It is involved in rapidly activating phosphorylated inositol (4,5) P2 (PIP2) to phosphorylated inositol (3,4,5) P3 (PI3P). Protein kinase B (p-Akt) is a downstream protein of PI3K that is recruited by PI3P and phosphorylated by phosphatidylinositol-dependent kinase (PDK-1) [39]. P-Akt can modulate the levels of phosphorylated mTOR proteins to promote cell proliferation and growth [40]. VEGF and TGF-β1 pathways are activated in response to tissue injury, encouraging fibroblasts to migrate to the wound and differentiate into myofibroblasts [41]. These cells may contribute to wound edge contraction and produce collagen-like proteins necessary for wound healing and remodeling, accelerating DTPI wound repair. The phosphorylated PI3K/Akt/mTOR pathway was significantly increased in the dECM-treated group compared to the control group, as shown by western blot analysis. This finding suggests that a primary mechanism by which the dECM hydrogel enhances DTPI repair may be the activation of the PI3K/Akt/mTOR signaling pathway.

In addition, PI3K-Akt-mTOR is the most critical signaling pathway for the regulation of autophagy [42, 43]. Moreover, mTOR promotes Akt phosphorylation, leading to mTOR reactivation or direct regulation of autophagy-repressing FOXO transcription factors [44]. Chronic wound healing disorders are associated with increased autophagy, an essential mechanism in regulating cellular functions, and have been closely linked to disease progression [45]. Previous studies have reported that advanced glycation end products (AGEs) can cause refractory wounds by promoting macrophage polarization toward the M1 phenotype through autophagy activation [46]. It is also possible to eliminate AGEs through p62-mediated autophagy [45]. In principle, autophagy can be regulated by the production of IGF-1 by the macrophages. IGF-1 is also produced by muscle cells during exercise. It may have a trophic and physiological role independent of initiating inflammation. Furthermore, IL-6 can affect the Akt/mTOR pathway and protein synthesis, which may influence autophagy [47]. Whether or not there is a correlation between the progression of DTPI and autophagy needs to be further validated.

## Conclusions

This study identified iron deposition and hyperactivation of M1 macrophages in developing DTPI. Then, we successfully prepared an injectable hydrogel from porcine skeletal dECM, ameliorating the iron deposition in the DTPI tissues through immunomodulation and enhancing M1–M2 macrophage transformation. In addition, the hydrogel induced a multidimensional coordinated tissue repair process by promoting vascular neovascularization and skeletal muscle regeneration. It is foreseeable dECM hydrogel derived from skeletal muscle is suitable for clinical translation due to its superior pro-regenerative ability, scalable manufacturing and easy administration. dECM hydrogel can also treat other muscle injury-related defective tissues or macrophage immune dysregulation-induced inflammatory diseases.

## Figures and Tables

**Table 1.** Sequences of gene primer for qRT–PCR^a^

Gene name	Primer sequences (5’→3’)	Amplicon length (bp)
iNOS	F: GCTGCCAGGGTCACAACTTTA	131
	R: CAGCTCAGTCCCTTCACCAA	
TNF-α	F: CCCTCACACTCACAAACCAC	205
	R: ACAAGGTACAACCCATCGGC	
Myh8	F: ATGATGTTTCACAGCTGCAGAG	102
	R: CCATCATGGCGGCATCAGTA	
Fech	F: GAAGCCCACCATGAAGTGGA	195
	R: ACCTCTTGGGGATAGGGGTC	
Fth1	F: CTGGAACTGCACAAACTGGC	200
	R: CTCTCATCACCGTGTCCCAG	
Myom3	F: CCTAGAGACTGCCCAGTGGA	192
	R: CTGCACGGAAGAACCCATCT	
Myo10	F: TTGGTTTGCTGGTGATCCGA	179
	R: AAGTTCCTCACTTCTGGGCG	
	F: CTTTTCTGTCCAGGCCGAGG	132
Myh14	R: TAGAGGAACGATTGGGCTGC	
CD163	F: TGCTGTCACTAACGCTCCTG	138
	R: TCATTCATGCTCCAGCCGTT	
IL-10	F: TTAAGGGTTACTTGGGTTGC	137
	R: GAGGGTCTTCAGCTTCTCAC	
Arg1	F: CCTGCTGGGAAGGAAGAAAAGG	162
	R: CCTGAAAGGAGCCCTGTCTTGTA	
Slc40a1	F: CAGACTTAAAGTGGCCCAGACG	139
	R: GTAGCAGACAGTAAGGACCCATC	
β-actin	F: AGGTCATCACTATTGGCAACGAG	151
	R: TTGGCATAGAGGTCTTTACGGAT	

**Note:** iNOS inducible Nitric Oxide Synthase, IL-10 interleukin-10, TNF-α tumor necrosis factor-α, Fth1 ferritin heavy chain 1, Myh8 myosin Heavy Chain 8, Myom3 myomesin-3, Myo10 myosin 10, Myh14 myosin heavy chain 14, Fech ferrochelatase, Arg1 arginase1, Slc40a1 solute carrier family 40, CD163.
